# Editorial: The key role of lipids in the regulation of ion channels

**DOI:** 10.3389/fphys.2022.1000082

**Published:** 2022-09-07

**Authors:** Andrea Saponaro, Marco Lolicato

**Affiliations:** ^1^ Department of Biosciences, University of Milano, Milano, Italy; ^2^ Department of Molecular Medicine, University of Pavia, Pavia, Italy

**Keywords:** lipids, Ion channels, regulation, biomembranes, biophysics

## Introduction

Biomembranes are a complex and dynamic environment, whose lipid composition influences the activity of membrane proteins. Lipid regulation of membrane proteins can be grouped in two general mechanisms: 1) direct regulation due to lipid interaction with proteins; 2) modification of the properties of the membranes (stiffness, stretches, compression, etc.), which in turns affects membrane protein activity.

Ion channels represent a crucial class of membrane proteins as they control the membrane potential in response to extracellular and intracellular stimuli. Indeed, a vast repertoire of cell signalling pathways, especially in excitable cells as neurons, originate from ion channels activity.

The understanding of the molecular mechanisms of the lipid-dependent regulation of ion channels is therefore crucial for the advancement in the comprehension of the physiological and pathological roles of this class of membrane proteins. The present Research Topic focuses on the cooperation between lipids and ion channels.

The recent advancements in membrane proteins structural biology by single particle cryo-EM, together with the significant support of native mass spectrometry and of molecular dynamics simulations, had greatly fostered the identification and characterization of lipid binding sites in the structures of ion channels. These findings have finally clarified the yet unknown molecular details of ion channel modulation by lipids, or even uncovered new mechanisms of channel regulation. In this context are inserted four studies of this Research Topic. Indeed, the research of Cabezudo et al. characterizes the atomic details of phosphoinositides interaction with hyperpolarization-activated cyclic-nucleotide gated (HCN) channels, while the work of Lummis et al. investigates the molecular determinant of cholesterol regulation of pentameric ligand-gated ion channels. Moreover, the two reviews of Kawai and Okamura and of Cheng et al. highlight the regulatory effect of lipid binding to ion channels from two distinct, but complementary points of view: 1) the modulation exerted by a particular lipid on a variety of ion channels; 2) the lipids involved in the control of the functionality of two specific family of channels.

Lipids can also influence ion channel function indirectly by modifying the physical properties of the membrane environment around the protein. Because of this, often, membrane proteins modify their microenvironment to maximize their functionality. Therefore, there is a mutual influence between the membrane environment and the protein residing in it. This crucial theme is also represented in the Research Topic with the two works of McGuire and Blunck and of Maer et al.

## Regulation of ion channels due to lipid—protein interaction.

Phosphoinositides (PIPs) comprise a minor proportion of the lipid membrane, but they play important roles in a variety of physiological processes, including signal transduction, regulation of cytoskeleton, exocytosis, and endocytosis (Balla, 2013). Cabezudo et al. studied, at atomic level, the interaction between phosphoinositides (PIPs), and hyperpolarization-activated cyclic-nucleotide gated (HCN) channels, a family of channels that plays a key role in controlling rhythmic activity in cardiac pacemaker cells and spontaneously firing neurons (Robinson and Siegelbaum, 2003). Specifically, PIPs have been shown to enhance HCN activation (Pian et al., 2007). Cabezudo et al., by employing computational approaches and the cryo-EM structure of HCN1 as a template (Lee and MacKinnon, 2017), show that PIP binding to HCN channels appear not to be well coordinated, as it occurs in several proteins, but rather involves a broad number of charged residues at the interface between the transmembrane region and the cytosolic machinery of the channel. Moreover, Cabezudo et al. show that phosphatidylinositol (PI) interactions with HCN1 structure possess a very low affinity. This finding provides an atomistic explanation of the fact that the unphosphorylated PI displays no effect on HCN1 activation, unlike phosphorylated PIPs (Pian et al., 2007). The work of Cabezudo et al. shows how fruitful is the combination of the recent advancements in structural biology (single particle cryo-EM) and *in silico* analysis (docking and molecular dynamics simulation) for the characterization of the association between lipids and ion channels and thus for the understanding of the regulation exerted by lipids.

Connected to the topic of the work of Cabezudo et al. is the review of Kawai and Okamura, as it pertains to the regulation exerted by PtdIns(4,5)P2, a specific PIP, on ion channels. Starting from their recent study of the relationship occurring between PtdIns(4,5)P2 and Slo3, a sperm specific K^+^ channel, in native cells (Kawai et al., 2019), Kawai and Okamura in the present review shed light on the current understanding about PtdIns(4,5)P2 affinity for diverse ion channels and their possible regulatory mechanism in physiological conditions. Indeed, even though PtdIns(4,5)P2 has been identified as the modulator of several tens of ion channels (Suh and Hille, 2008; Okamura et al., 2018), the regulation exerted by the lipid was characterized by using a synthetic version of it, which may not fully account for its real modulatory effects. On the contrary, few studies have been characterized the role of PtdIdns(4,5)P2 in native environments. Among these there is the recent work (Kawai et al., 2019) of the authors of the review. This is a key point to understand the importance of the mini-review of Kawai and Okamura. First, the authors summarize the state of the art concerning the list of ion channels known to be target of PtdIns(4,5)P2 and how their sensitivity for this PIP has been characterized in heterologous expression system. In the second part of the review, the authors raise the possibility that a way of regulating/compensating the large variability in the affinity of ion channels for PtdIns(4,5)P2 may be represented by their compartmentalization in regions possessing a given concentration of PtdIns(4,5)P2. This hypothesis is based on a recent discovery of the authors of the review showing that in the membrane of the sperm flagellum Slo3 channel, a high-affinity binder of PtdIns(4,5)P2, localizes in regions where the density of PtdIns(4,5)P2 is extremely low and that this most likely represent a way of compensating for its high-affinity to the lipid (Kawai et al., 2019). In agreement with this view, it is known that the levels of PtdIns(4,5)P2 are cell type dependent. PtdIns(4,5)P2 is, for instance, enriched in dendritic spines of cultured hippocampal neurons (Horne and Dell’Acqua, 2007). On the contrary, Kawai and Okamura reported very low level of PtdIns(4,5)P2 in the sperm flagellum (Kawai et al., 2019). Strikingly, PtdIns(4,5)P2 levels can also be extremely dynamic: It has been reported that they vary during cell-division cycle (Phua et al., 2017). Therefore, the activity of the ion channels that are targets of PtdIns(4,5)P2 can be dynamically modulate based on the membrane environment. This concept, explored in the review for Slo3 and for the ion channels regulated by PtdIns(4,5)P2, can be easily become a general rule of membrane proteins.

Another well-known example of lipids directing controlling ion channel function concerns the pentameric ligand-gated (pLGIC) channels, whose gating is modulated by the direct interactions of lipids with their outermost lipid-facing α-helix M4, also known as the lipid sensor of this family of channels (Salari et al., 2014). The study of Lummis et al. further highlights the key role of M4 in lipid binding and channel modulation starting from the apparent discrepancies in the biophysical properties displayed by α4β2 nACh receptor, the major nACh receptor subtypes in the human brain, when expressed in different model systems. In particular, the authors discover that several mutations in the α4β2 nACh receptor previously classified as “loss of function” when expressed in mammalian cells (Mesoy and Lummis, 2021), in Xenopus oocytes do not impair receptor activity. Moreover, Lummis et al. show that several mutations known to modify channel gating when expressed in mammalian cells, do not behave differently from wild-type in oocytes. Based on the cryo-EM structure of α4β2 nACh receptor, where cholesterol molecules were identified in a “bowl-shaped” surface formed at the interface between M3, MX and M4 helices of two adjacent protomers (Walsh et al., 2018), the authors suggested that a different lipid content, in particular cholesterol, of the plasma membrane of HEK cells and oocytes is at the basis of the discrepancies. Strikingly, most of the mutations are in the cholesterol binding surface, or nearby. Thus, the authors indicate cholesterol as the major candidate to explain the differences functionality α4β2 nACh mutants. Indeed, in a low cholesterol environment the mutant receptors could be in a lipid uncoupled form, leading to a non-functional state. In well agreement with this hypothesis, it has been reported that the membranes of HEK cell have a lower cholesterol:lipid ratio than those of Xenopus oocyte (Opekarová and Tanner, 2003; Dawaliby et al., 2016). Lummis et al. frame their findings on nACh receptor in the physiological context of dynamics of synapses. Indeed, the compartmentalization of the receptors into different membrane microenvironments may represent a cellular procedure to obtain a large modulation of the activity of nACh receptor. This agrees with the model of regulation of ion channels based on the compartmentalization proposed in the review of Kawai and Okamura. Moreover, the work of Lummis et al. draws attention on the limit of model systems, which may not always accurately reproduce the physiological contexts where ion channels reside.

Finally, the review of Cheng et al. provides a different, but complementary perspective compared to the previously described works as it addresses the issue of lipid regulation of ion channels from the point of view of pharmacology. Cheng et al., indeed, highlight the key contribution of the cryo-EM structures of eukaryotic ion channels in the understanding, at atomic details, of the mechanism of lipid regulation via lipid-protein interactions, or in the identification of novel lipid binding sites. Moreover, the authors pointed out the striking finding that many of these lipid binding sites are well-known sites for allosteric modulators or drugs. This finding supports the idea that lipids may act as endogenous allosteric modulators of ion channels. Cheng et al. focus their review on the shared binding sites between lipids and drugs and use the pentameric ligand-gated ion channels and transient receptor potential channels as examples. The authors discuss the intriguing hypothesis that lipids may alter drug potency, as well as that the characterization at atomic level of lipid binding pockets is a powerful starting point to guide drug design. Moreover, Cheng et al. highlight that, though the cryo-EM structures are a milestone in the research field of ion channels, they represent only a part of the process leading to the understanding of the mechanism of lipid regulation. In this light, the authors describe complementary approaches employed to fill the gaps in the knowledge of lipid-protein interaction like, for instance, the use of native mass spectrometry for the identification of lipid binding sites and their affinities for lipids; characterization of ion channel function in reconstituted systems using asymmetric membranes; fluorescence binding assays with tryptophan fluorescence quenching by brominated lipids; computational approaches. This review well describes the current state of the art methodologies employed in the field to unravel the role of lipid binding and channel regulation.

## Indirect modulation of ion channels *via* changes of the membrane properties

The chemical and physical properties of the membrane environment play a pivotal role in regulating membrane protein function, which, indeed, modify the surrounding to their advantage. In this light, the work of McGuire and Blunck significantly contributed to the advancement of the filed as they were able 1) to demonstrate that the prokaryotic potassium channel KcsA modifies the microenvironment around it and 2) to connect the functionality of KcsA with the modifications of the membrane induced by the protein. By simultaneously imaging clustering of KcsA molecules in synthetic planar lipid bilayers and recording single channel opening, McGuire and Blunck demonstrated that clustering, which coincides with cooperative opening of KcsA (Molina et al., 2006; Sumino et al., 2014), is driven by the negative curvatures of the microenvironment around the channel. It is worth noting that this work clarifies a long-lasting dispute on the mechanism of clustering of KcsA. Indeed, McGuire and Blunck demonstrate that clustering is not caused by direct protein-protein interactions (Visscher et al., 2017) or hydrophobic mismatch with the lipid environment (Williamson et al., 2003), but rather driven by an invagination of the lipid matrix around the channel, induced by the protein.

The mutual influence between the membrane curvature and membrane proteins, as well as the concept of membrane curvature as part of the mechanism of regulation of membrane proteins has been consolidated over time. Milestone examples of this are, for instance, the membrane curvature of the tubular cristae of mitochondria induced by the dimerization of the ATP Synthase (Blum et al., 2019), the proposed gating mechanism of mechanosensitive Piezo channels (Guo and MacKinnon, 2017) and of BKCa channels (Blunck et al., 2001; Crowley et al., 2005) and the influence of the membrane curvature in the photoactivation of Rhodopsins (Soubias et al., 2010). The work of McGuire and Blunck not only corroborates the theory of the mutual influence between membrane proteins, their functionality and the microenvironment surrounding them, but also proposes a single molecule methodology to expand this characterization to a wide range of membrane proteins.

In line with McGuire and Blunck, the work of Maer et al. highlights the key role of the lipid intrinsic curvature (c_0_) in the regulation of membrane protein function. To do so, they tested how different experimental manoeuvres, which alter the effective size of phospholipid head groups, affect the functionality of Gramicidin Channels. Overall, they discovered that the alterations in the size of the polar head groups induced by the manoeuvres modulate Gramicidin channel function as would be predicted from the expected changes in c_0_. Of note that, since the interaction between the head groups of lipids can alter bilayer properties other than c0, such as thickness or elastic moduli, the authors excluded a primary involvement of the latter properties in the regulation of gramicidin function as they demonstrated that their changes are too small to have measurable effects on the electrical activity Gramicidin. Therefore, Maer et al. highlight the primary role of the alteration of the lipid curvature around Gramicidin channel for its regulation.

Summarizing, the articles in this Research Topic offer a wealth of insight into the two main mechanisms of regulation of ion channels exerted by lipids: 1) direct interaction with their target proteins; 2) indirect modulation of the surrounding environment of their targets. The articles propose advanced methodologies to characterize, from the atomic to the cellular level, the mechanisms of regulation of ion channels exerted by lipids, as well as to identify novel putative lipids binding sites and prove them biochemically and functionally.
